# Gene Expression Profiling associated with Hepatoxicity in Pregnant Rats treated with Ubi Gadong (*Dioscorea hispida*) Extract

**Published:** 2017-03

**Authors:** Ezarul Faradianna Lokman, Hussin Muhammad, Norizah Awang, Maizatul Hasyima Omar, Fazliana Mansor, Fatin Saparuddin

**Affiliations:** 1 Diabetes and Endocrine Unit, Cardiovascular, Diabetes and Nutrition Research Centre (CDNRC);; 2 Toxicology and Pharmacology Unit, Herbal Medicine Research Centre (HMRC);; 3 Phytochemistry Unit, Herbal Medicine Research Centre (HMRC), Institute for Medical Research, Jalan Pahang, 50588, Kuala Lumpur, Malaysia

**Keywords:** hepatoxicity, Dioscorea hispida, pregnant rat, gene expression

## Abstract

*Dioscorea hispida* (*D.hispida*) is the most well-known starchy tuber in Malaysia and called ‘ubi gadong’. Despite concerns over toxicity effects, the tuber is known to possess therapeutic values due to the presence of bioactive compounds such as saponins. This study was performed to identify the changes in gene expression profiles associated with hepatoxicity in pregnant rat treated with *D.hispida* using RT² Profiler PCR Array. The identification of steroidal saponins from *D.hispida* was carried out by UHPLC/MS method. Treatment of *D.hispida* caused mortality when dosage above 2000 mg/kg b.w. was given to pregnant rats. The PCR array showed that several genes were significantly up and down-regulated upon treatment with *D.hispida*. Treatment of *D.hispida* at 2000 mg/kg b.w leads to significant upregulation of several genes such as Btg2, Gsr, L2hgdn, S100a8, Slc17a3, Bhmt, Cd68, Cyp1a2 whereas several genes were downregulated such as Abcb1a, Aldoa, Cdc14b, Icam1, Krt18, Hpn and Maob. The consumption of *D.hispida* extract when taken at lower dosage of 2000 mg/kg may not be harmful to rats. *D.hispida* extract given at the highest dosage to pregnant rats caused alterations of several genes categorized in different hepatotoxic group functions such as necrosis, cholestasis and phospholipodisis.

## INTRODUCTION

*Dioscorea* (Family *Dioscoreacea*) commonly known as yams, are staple foods for Africa, tropical America and Asia. Yams have been traditional consumed as starchy staple food (such as *Dioscorea opposita, Dioscorea alata, Dioscorea japonica*, and *Dioscorea hispida*). The genus *Dioscorea* L. consists of about 630 species distributed all over the world. *Dioscorea hispida* is the most well-known starchy tuber in Malaysia and it is called `ubi gadong’. Majority of this species contain steroidal saponins with potent pharmacologic activities. Some of *Dioscorea* species being the source of the steroidal sapogenin, diosgenin a precursor for the synthesis of steroidal drugs. The glycoside form of diosgenin is called Dioscin (1).

Many of the yam tubers are reported to be toxic containing cyanides and have to be detoxified by soaking in flowing water and cooking before consumption in order to remove the toxic compound presents in them (2, 3). However, since traditional detoxified methods are difficult to practice, new dioscorine detoxified equipment was developed to improve the quality and time of detoxified process (4, 5). In terms of therapeutic values, several pharmacological studies have demonstrated that *Dioscin* exhibits anti-tumor (6), anti-hyperlipidemia (7), and anti-diabetic (8) properties. In addition, *D. hispida* extract exhibited significant anti-inflammatory and analgesic properties (9). The protective effect of *D. alata* however has also been shown previously in aniline-induced splenic toxicity rats due to its antioxidant property and the presence of different phytochemicals (10). *D. alata* contained alkaloid, flavanoid, saponin, tannin and phenol (11). In addition, phytochemistry and therapeutic potential of *Dioscorea bulbifera* have been discussed and used in different parts of the world as traditional medicine against several diseases including diabetes, digestive problems, cancer, inflammation and others (12).

Hepatoxocity is one of the most reported adverse effects caused by herbal products and mechanisms of action leading to toxicity due to the chemical complexity and mixtures of herbal compositions remain vague (13). *D. hispida* previouslycaused several poisoning symptoms, irregularities and complications to various organs including liver, spleen and kidney in rats(14, 15). *Benincasa hispida* aqueous ethanolic extract on the other hand showed survival in both acute and sub-acute toxicity studies in rodents and contain potential therapeutic values (16). However, to date, alterations of gene expression profiling affecting the liver tissue treated with *D.hispida* have not yet been performed mainly in pregnant rats. Overconsumption of this plant may promote health complications resulting in tissue damage particularly in the liver. This study was carried out to identify the effects of different concentrations of *D.hispida* on liver tissue and to identify the gene expression profiles in pregnant rats using PCR array analysis technique. The identification of steroidal saponins from *D.hispida* have been determined by UHPLC/MS. This information was then used to determine which metabolic and signal transduction pathways were affected following the mechanisms of action involved.

## MATERIALS AND METHODS

### Chemical

The steroidal saponins dioscin, protodioscin and prodeltonin were purchased from Chromadex, USA. Acetonitrile and formic acid LCMS grade was purchased from Fisher Scientific (M) Sdn. Bhd (Kuala Lumpur, Malaysia), reverse osmosis Milli-Q water (18.2 MΩ) (Millipore, Billerica, USA) was used for all solutions and dilutions.

### Plant samples

Fresh rhizomes and roots of *D. hispida* were obtained from Machang, Kelantan. Samples were washed, dried and grinded to a powdered form prior extraction with water. Prior to injection (10 mg/mL of dry extract), an adequate volume (ca. 2 mL) was passed through a 0.22 µm PTFE membrane filter.

### Analysis of steroidal saponins by UHPLC

The liquid chromatographic system was a QExactive UHPLC (Thermo Fischer Scientific, USA) comprised of the following components: binary pump, a solvent degasser, an autosampler, and a thermostatically controlled column compartment. Separation was achieved on WATERS C18 (2.1 × 50 mm, 17 µm) column. The mobile phase consisted of water with 0.1% formic acid (A) and acetonitrile with 0.1% formic acid (B) at flow rate 0.4 mL/min. The following solvent composition was used: 0-5 min, 10-20% B (linear gradient); 5-10 min, 20-60% B (linear gradient); 10-13 min, 60-90 % B (linear gradient); 14-14.10 min, 90-10 % B (linear gradient) and 14.1-15 min, 10% B (isocratic).

### Animals

For toxicity study, healthy virgin females and confirmed fertile males (2:1) Sprague Dawley rats with a body weight ranging from 180-200 g and 200-250 g, respectively were obtained from the Animal Resource Unit, Medical Resource Centre, Institute for Medical Research. The animals were housed in polypropylene cages, lined with wood shaving and at controlled temperature (20 ± 2°C), with 40-60% humidity under 12 hours of light and dark cycle. The animals were acclimatized for about a week prior to start of the study. A commercial rat diet (Specialty Feeds, Australia) and water were available *ad libitum*. Ethical approval for this study was obtained from the Animal Care and Ethics Committee, Ministry of Health Malaysia (ACUC No: ACUC/KKM/02 (10/2016).

### Experimental design

Female rats in the proestrus phase were placed into the cage of males (1:1 basis) in the late morning and left for 24 hours. The vaginal smear was performed on each rat. The mating was confirmed by the presence of sperm and designated as gestation day (GD) 0.

### Treatment

The dams were randomly assigned to four treatment groups (Group 1: 250, Group 2: 1000 and Group 3: 2000 mg/kg body weight) and one control group (distilled water) and caged individually. For gene expression analysis, 3 rats were selected from each group. The dams were administered with *D.hispida *aqueous extract once daily by gastric gavage on GD6 to GD20. On GD21 rats were euthanized by carbon dioxide inhalation and caesarean hysterectomy was immediately performed. The liver was removed and immediately stored in RNA later. The samples were kept at -80°C until RNA extraction.

### RNA extraction

A total of 30 mg of each liver tissue was disrupted using a Tissue Ruptor (230V, 50-60Hz, UK) attached with a probe in 600 µl of 1:100 beta-mercaptoethanol in RLT buffer. The samples were then centrifuged for 3 min. The supernatant was collected and mixed with 1 volume of 70% ethanol. All samples were then further processed for total RNA using RNeasy Total RNA Mini Kit (Qiagen) using manufacturer recommended procedures. RNA concentration was measured using Multiskan GO Microplate Spectrophotometer (Thermoscientific, USA).

### PCR array analysis

Gene expression analysis was performed using the PARN 093Z RT^2^ Profiler PCR Array Rat Hepatotoxicity System and Protocol Guide. The Rat Hepatotoxicity PCR Array profiles the expression of 84 key genes implicated as potential biomarkers of liver toxicity. Initially, 100 ng of total RNA sample was converted to cDNA using RT^2^ PreAMP cDNA Synthesis Kit. cDNA was added to RT^2^ qPCR Master Mix and aliquoted across PCR array. The samples were run using Real Time PCR Instrument (StepOnePlus, ABI Biosystem). RT^2^ PCR Data Analysis Software was used for data analysis. Data normalization step was performed. Three housekeeping genes were selected in normalization step known as B2m, Hprt1 and Rplp1 genes. Fold-regulation represents fold-change results in a biologically meaningful way. Fold-change values greater than one indicates a positive- or an up-regulation, and the fold-regulation is equal to the fold-change. Fold-change values less than one indicate a negative or down-regulation, and the fold-regulation is the negative inverse of the fold-change. The p values are calculated based on a Student’s t-test of the replicate 2^∧^ (- Delta CT) values for each gene in the control group and treatment groups. The cut-off points for gene array analysis were >2 fold change with p-value <0.05.

## RESULTS

**Figure 1:**
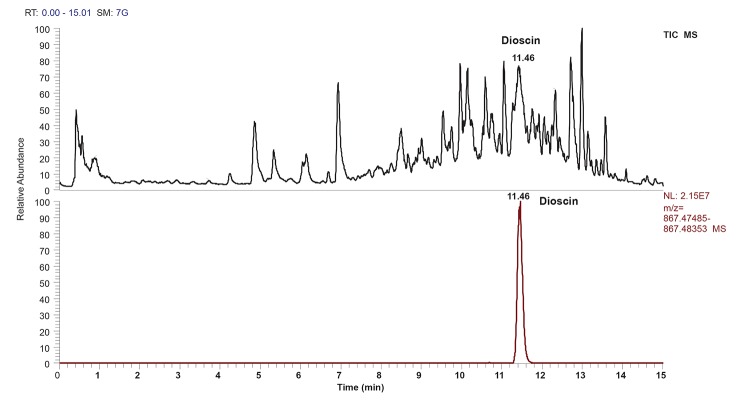
Typical UPLC Total ion chromatography (TIC) of plant extract.

**Figure 2:**
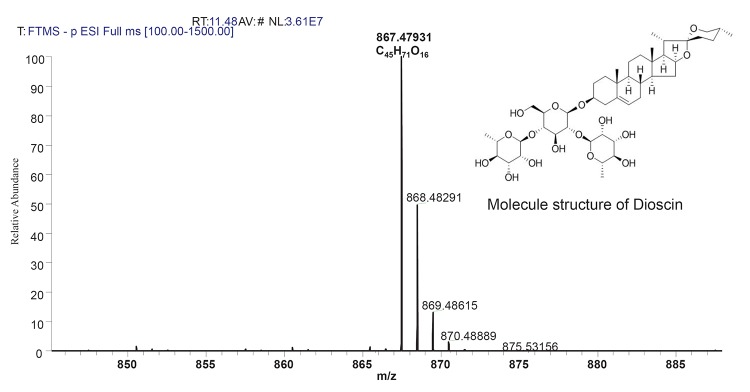
Chemical structure and MS spectrum of dioscin in negative ion mode.

Ultrahigh performance liquid chromatography liquid chromatography (UHPLC) with MS detection has been used as an efficient tool for sample analysis and to facilitate structural elucidation. In the present study, mass specific detection was employed for identification of steroidal saponin due to its lack of specific chromophore for UV/VIS. Identification was performed using UHPLC-ESI-MS under developed chromatographic condition (Figure 1). Compound with a retention time of 11.48 min, produced a strong deprotonated molecule [M-H]- (m/z 867.47931) in (-)-ESI-MS. This compound is the main steroidal saponin present in the tuber and was tentatively identified as dioscin with structure molecule as shown in Figure 2 and Table 1. Rat Hepatotoxicity RT² Profiler™ PCR Array consists of the most relevant potential biomarkers for five major drug-induced hepatotoxic diseases such as cholestasis, steatosis, phospholipidosis, non-genotoxic hepatocarcinogenicity and necrosis, as well as generalized hepatotoxicity. Using real-time PCR, the expression of selected genes involved in hepatotoxic response with this array can be analyzed. Genes that consistently exhibit increased or decreased expression during these toxic responses in model systems serve as markers to predict potential adverse clinical outcomes.

**Table 1:** Mass spectral characteristic of Dioscin

Retention time (min)	Compound	Formula	Selected ion	m/z calculated	m/z observed	Error (ppm)

11.53	Dioscin	C45H71O16	[M-H]-	869.4899	867.47919	6.303

No significant difference was observed at 250 and 1000 mg/kg b.w of *D. hispida* as compared to the control group (Table 2). However, several genes were significantly down and up-regulated upon treatment with *D.hispida* given at the highest dosage of 2000 mg/kg. Treatment of *D.hispida* at 2000 mg/kg b.w leads to significant upregulation of several genes such as Btg2 (32.22;0.02), Gsr (41.83;0.02), L2hgdn (20.52;0.05), S100a8 (51.28;0.03), Slc17a3 (2.24;0.02), Bhmt (1.85; 0.05), Cd68 (2.19; 0.02), Cyp1a2 (19.91;0.05) whereas several genes were downregulated such as Abcb1a (-29.31; 0.01), Aldoa (-3.45;0.05), Cdc14b (-4.93;0.03), Icam1 (-30.36; 0.02), Krt18 (-5.41; 0.03), Hpn (-4.33; 0.01) and Maob (-2.52; 0.04).

**Table 2:** Fold regulation comparing to the Control group at different concentration of *D. hispida*

Gene Symbol	Description	Fold Regulation (comparing to control group)					
		mg/kg					
		250		1000		2000	
		Fold Regulation	*p*-value	Fold Regulation	*p*-value	Fold Regulation	*p*-value

Abcb11	ATP-binding cassette, subfamily B (MDR/TAP), member 11	0.00	0.95	-1.83	0.51	4.38	0.38
Abcb1a	ATP-binding cassette, sub-family B (MDR/TAP), member 1A	-2.49	0.51	-1.27	0.39	-29.31	0.01
Abcb4	ATP-binding cassette, subfamily B (MDR/TAP), member 4	1.11	0.66	-1.09	0.39	-2.02	0.90
Abcc2	ATP-binding cassette, subfamily C (CFTR/MRP), member 2	-1.32	0.81	1.74	0.39	1.22	0.36
Abcc3	ATP-binding cassette, subfamily C (CFTR/MRP), member 3	1.50	0.47	3.51	0.38	-1.50	0.67
Aldoa	Aldolase A, fructose-bisphosphate	1.14	0.83	6.69	0.38	-3.45	0.05
Apex1	APEX nuclease (multifunctional DNA repair enzyme) 1	2.13	0.69	8.25	0.37	-1.70	0.53
Asah1	N-acylsphingosine amidohydrolase (acid ceramidase) 1	1.17	0.62	6.86	0.38	-1.18	0.57
Atp8b1	ATPase, Class I, type 8B, member 1	1.28	0.74	15.84	0.37	-1.39	0.57
Avpr1a	Arginine vasopressin receptor 1A	1.35	0.71	25.27	0.37	-1.10	0.73
Bhmt	Betaine-homocysteine methyltransferase	-1.28	0.78	7.70	0.37	1.85	0.05
Btg2	BTG family, member 2	8.78	0.10	2.65	0.36	32.22	0.02
Car3	Carbonic anhydrase 3	-11.84	0.24	-46.93	0.18	-4.90	0.84
Casp3	Caspase 3	-1.23	0.36	-4.09	0.54	1.78	0.40
Ccng1	Cyclin G1	-2.77	0.16	-31.21	0.12	-10.71	0.28
Cd36	CD36 molecule (thrombospondin receptor)	3.27	0.17	13.28	0.37	4.33	0.25
Cd68	Cd68 molecule	1.21	0.79	10.98	0.37	2.19	0.02
Cdc14b	CDC14 cell division cycle 14 homolog B (S. cerevisiae)	-1.36	0.55	4.68	0.38	-4.93	0.03
Cdkn1a	Cyclin-dependent kinase inhibitor 1A	-2.08	0.45	3.79	0.38	-6.09	0.09
Col4a1	Collagen, type IV, alpha 1	-1.07	0.43	9.16	0.39	-1.58	0.42
Cryl1	Crystallin, lambda 1	-1.81	0.41	5.18	0.39	-1.96	0.43
Cxcl12	Chemokine (C-X-C motif) ligand 12 (stromal cell-derived factor 1)	-1.36	0.48	6.18	0.38	-1.46	0.66
Cyp1a2	Cytochrome P450, family 1, subfamily a, polypeptide 2	19.91	0.05	18.94	0.37	19.91	0.05
Ddit4l2	DNA-damage-inducible transcript 4-like	-2.77	0.55	1.61	0.38	-1.85	0.57
Ddx39a	DEAD (Asp-Glu-Ala-Asp) box polypeptide 39	-6.01	0.22	-52.24	0.13	-1.29	0.44
Dnajb11	DnaJ (Hsp40) homolog, subfamily B, member 11	-1.27	0.18	-13.59	0.15	2.81	0.38
Dnajc3	DnaJ (Hsp40) homolog, subfamily C, member 3	-2.82	0.09	-8.13	0.48	-8.23	0.61
Fabp1	Fatty acid binding protein 1, liver	-1.09	0.82	6.22	0.37	2.00	0.22
Fads1	Fatty acid desaturase 1	-1.24	0.66	6.57	0.38	-2.85	0.63
Emc9	Family with sequence similarity 158, member A	-1.22	0.55	7.03	0.37	-5.71	0.15
Fasn	Fatty acid synthase	-1.87	0.41	6.07	0.38	-2.87	0.39
Fmo1	Flavin containing monooxygenase 1	27.15	0.37	4.60	0.39	-3.16	0.46
Timm10b	Fractured callus expressed transcript 1	-1.57	0.44	7.27	0.38	-1.33	0.59
Gadd45a	Growth arrest and DNA-damage-inducible, alpha	1186.49	0.37	5686.80	0.37	18.32	0.23
Gclc	Glutamate-cysteine ligase, catalytic subunit	-3.28	0.44	-11.74	0.23	7.17	0.42
Gsr	Glutathione reductase	9.75	0.34	4.31	0.37	41.83	0.02
Hao2	Hydroxyacid oxidase 2 (long chain)	-46.98	0.13	-94.31	0.13	-2.92	0.48
Hmox1	Heme oxygenase (decycling) 1	-1.11	0.45	-3.51	0.71	-1.04	0.46
Hpn	Hepsin	-4.33	0.01	-3.29	0.40	-4.33	0.01
Hyou1	Hypoxia up-regulated 1	-1.34	0.53	4.31	0.38	-1.07	0.61
Icam1	Intercellular adhesion molecule 1	35.07	0.37	-1.32	0.40	-30.36	0.02
Igfals	Insulin-like growth factor binding protein, acid labile subunit	-2.55	0.35	4.56	0.38	-4.01	0.33
Il6st	Interleukin 6 signal transducer	-1.53	0.42	5.06	0.39	-2.86	0.39
Ipo4	Importin 4	-1.02	0.50	14.04	0.38	-1.33	0.49
Krt18	Keratin 18	-4.43	0.17	4.26	0.38	-5.41	0.03
Krt8	Keratin 8	8.14	0.17	107.66	0.37	13.66	0.20
L2hgdh	L-2-hydroxyglutarate dehydrogenase	4.78	0.36	4.19	0.39	20.52	0.05
Lgr5	Leucine rich repeat containing G protein coupled receptor 5	-2.98	0.31	-1.13	0.49	-2.89	0.41
Lpl	Lipoprotein lipase	-47.23	0.18	1.09	0.38	2.02	0.44
Lss	Lanosterol synthase (2,3-oxidosqualene-lanosterol cyclase)	-2.65	0.14	-3.42	0.91	-1.12	0.45
Maob	Monoamine oxidase B	-2.52	0.04	-5.19	0.57	-2.52	0.04
Map3k6	Mitogen-activated protein kinase kinase kinase 6	1.47	0.54	8.81	0.37	-1.05	0.75
Mbl2	Mannose-binding lectin (protein C) 2	1.10	0.86	4.25	0.38	-12.46	0.43
Mcm10	Minichromosome maintenance complex component 10	151.55	0.37	-1.26	0.98	-3.90	0.12
Mlxipl	MLX interacting protein-like	19.18	0.37	8.29	0.38	-2.60	0.43
Mrps18b	Mitochondrial ribosomal protein S18B	1.03	0.43	18.50	0.38	1.13	0.45
Nqo1	NAD(P)H dehydrogenase, quinone 1	-1.08	0.60	10.09	0.37	-2.07	0.41
Nus1	Nuclear undecaprenyl pyrophosphate synthase 1 homolog (S. cerevisiae)	1.10	0.89	11.43	0.36	-1.16	0.85
Osmr	Oncostatin M receptor	27.38	0.21	411.08	0.35	13.46	0.17
Slc51a	Organic solute transporter alpha	1.72	0.45	1.47	0.57	-1.00	0.77
Pdyn	Prodynorphin	-4.09	0.23	2.53	0.40	-3.41	0.30
Pla2g12a	Phospholipase A2, group XIIA	3.09	0.13	-4.72	0.87	5.18	0.27
Ppara	Peroxisome proliferator activated receptor alpha	-1.66	0.44	1.11	0.48	-3.79	0.10
Psme3	Proteasome (prosome, macropain) activator subunit 3	1.57	0.29	10.78	0.37	1.17	0.43
Pygl	Phosphorylase, glycogen, liver	-1.53	0.47	2.84	0.38	-10.45	0.31
Rb1	Retinoblastoma 1	1.69	0.75	27.04	0.37	-1.92	0.42
Rdx	Radixin	-1.38	0.46	9.09	0.38	-1.97	0.44
Fam214a	Similar to hypothetical protein MGC38960	7.34	0.38	9.01	0.38	-2.10	0.44
Rhbg	Rh family, B glycoprotein	-1.26	0.44	8.17	0.38	-1.73	0.45
S100a8	S100 calcium binding protein A8	11.95	0.08	159.15	0.37	51.28	0.03
Scd1	Stearoyl-Coenzyme A desaturase 1	14.10	0.55	209.90	0.36	17.73	0.41
Serpina3n	Serine (or cysteine) peptidase inhibitor, clade A, member 3N	252.30	0.14	509.34	0.36	33.40	0.25
Serpine1	Serpin peptidase inhibitor, clade E (nexin, plasminogen activator inhibitor type 1), member 1	1.06	0.72	55.29	0.37	1.25	0.56
Skil	SKI-like oncogene	2.35	0.66	-5.11	0.35	2.50	0.52
Slc17a3	Solute carrier family 17 (sodium phosphate), member 3	-1.92	0.27	1.99	0.23	2.24	0.02
Slc2a3	Solute carrier family 2 (facilitated glucose transporter), member 3	-2.60	0.85	13.06	0.37	-1.87	0.68
Slc39a6	Solute carrier family 39 (zinc transporter), member 6	1.55	0.86	2.13	0.38	-7.38	0.31
Srebf1	Sterol regulatory element binding transcription factor 1	30.49	0.25	33.96	0.16	1.23	0.61
Tagln	Transgelin	-1.17	0.46	28.99	0.37	-1.51	0.47
Thrsp	Thyroid hormone responsive	-1.99	0.40	19.73	0.38	-4.85	0.38
Tmem2	Transmembrane protein 2	-1.09	0.46	-3.27	0.41	-2.10	0.41
Txnrd1	Thioredoxin reductase 1	3.72	0.14	80.77	0.37	2.95	0.33
Wipi1	WD repeat domain, phosphoinositide interacting 1	13.15	0.14	292.81	0.37	5.00	0.28
Yrdc	YrdC domain containing (E.coli)	17.37	0.06	42.69	0.33	12.14	0.19

## DISCUSSION

Our study showed that the highest dosage of 2000 mg/kg *D.hispida* containing an active compound known as Dioscin caused mortality to the pregnant rats. The PCR Array indicated that several genes were found to be significantly upregulated and downregulated as the concentration of *D.hispida* given to the rats increased. This could possibly explain that the regulations of several gene expressions were affected by different concentrations of *D.hispida* given.

Genes such as Bhmt, Gsr, Slc17a3, Btg2 and Cyp1a2 were upregulated and based on the functional groups, these genes are classified in hepatoxicity group. Cd68 and L2hgdh are known to be involved in necrosis. S100a8 is categorized in phospholipidosis. In addition, several genes were significantly downregulated at the highest dosage and classified into different functional groups; Abcb1a and Icam1 are associated with cholestasis. Hpn is involved in phospholipidosis whereas Maob is associated with hepatoxicity. Aldoa and Krt18 are associated with nongenotoxic hepatocarcinogenicity. Cdc14b is involved in necrosis.

The presence of Dioscin in *D.hispida* may be associated with hepatoprotective effects as shown in previous studies. Dioscin has been shown to have a good protective effect against APAP-induced liver injury and this protective action is related to the regulation of mitochondrial function (17). In another study, Dioscin exhibited potent effects against liver fibrosis through the modulation of multiple targets and signaling pathways (18).

Plants containing active compounds such as glucosides, acids or alkaloids are used as medicines, however when taken in excess may lead to several adverse effects (19). A previous study indicated that administration of *D. hispida* given up to 1250 mg/kg crude extract in rats caused several poisoning symptoms, irregularities and complications to various organs including liver and kidney (15). However, in another study involving acute and sub-acute toxicity experiments, 50% of *Benincasa hispida* aqueous ethanolic extract given up to 2000 mg/kg to rodents did not cause mortality, behaviour changes and no significant alteration in hematological as well as biochemical parameters results indicating that there is a wide margin of safety for the therapeutic use (16).

The knowledge of gene expression patterns found in this study has demonstrated practical benefits in terms of predicting pathological events and toxic endpoints. Thus, gene expression data can provide an early indication of toxicity because toxin-mediated changes in gene expression are often detectable before clinical observation, clinical chemistry or histopathology (20–22).

## CONCLUSIONS

The consumption of *D.hispida* extract containing Dioscin at lower dosage below 2000 mg/kg may not be harmful. In response to administration of *D.hispida* extract given at the highest dosage, the alterations of several genes categorized in different hepatotoxic group functions were observed affecting the liver functions of pregnant rats. The hepatoxicity gene array can be used in developing mechanism-based biomarkers for diagnosis or prognosis of hepatotoxicity-related coagulation abnormalities in the early stage of drug development.
